# Commentary: Big Brother is watching: Is there value in what is seen?

**DOI:** 10.1016/j.xjon.2021.10.010

**Published:** 2021-10-22

**Authors:** Kevin P. Landolfo, Rohan Goswami

**Affiliations:** a Department of Cardiothoracic Surgery, Mayo Clinic School of Medicine, Mayo Clinic Florida, Jacksonville, Fla; b Department of Transplant, Mayo Clinic School of Medicine, Mayo Clinic Florida, Jacksonville, Fla

**Figure:**
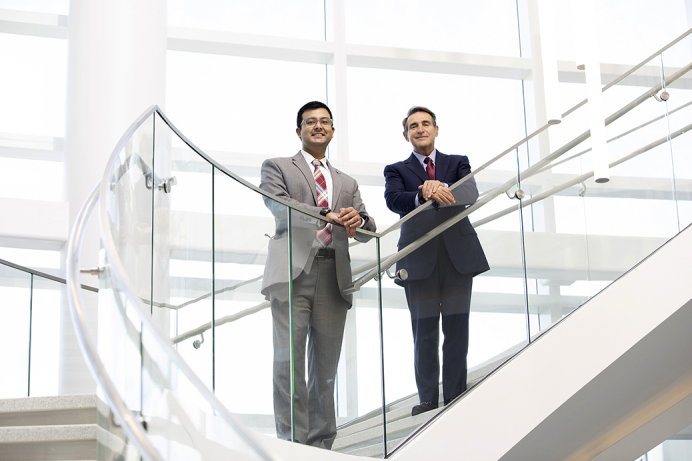
Rohan Goswami, MD, and Kevin P. Landolfo, MD, MSc

**Central Message**

Invasive hemodynamic assessment of patients with an LVAD may improve readmission rates, but early detection of nonresponders-to-therapy can lead to earlier LVAD therapy and transplant listing.

See Article page 18.

Left ventricular assist devices (LVADs) constitute important advanced therapy for patients with heart failure. Despite improvements in LVAD technology, optimal hemodynamic management remains challenging. In addition, frequent readmission, hemocompatibility-related adverse events, and thromboembolic complications limit the effectiveness of LVAD therapy.^1^, ^2^, ^3^

The development of implantable hemodynamic monitoring (IHM) allows continuous, remote measurement of pulmonary artery pressure, which is known to improve outcomes in patients with heart failure.^4^ Lambert and Teuteberg^5^ provide an expert review of IHM use in patients with an LVAD. The authors underscore the potential role of IHM to allow early identification of patients with refractory heart failure. Failure of pulmonary pressure to respond to guideline-directed medical therapy allows timely referral for LVAD consideration and improved outcomes in patients undergoing LVAD implantation with lower Interagency Registry for Mechanically Assisted Circulatory Support scores.^6^

Utilization of IHM technology for ongoing monitoring and optimization of LVAD therapy in the outpatient setting is reviewed. A patient vignette describes IHM use in a patient before and after LVAD therapy to demonstrate improvement in pulmonary artery pressures after LVAD implantation, thereby allowing cardiac transplant listing. However, limited evidence to date does not support a recommendation for routine use of IHM in patients with an LVAD. Further study of IHM in this patient population is suggested.

Current era LVAD complications that limit transplant potential include pulmonary hypertension (ie, refractory pulmonary vascular resistance) but are increasingly related to gastrointestinal bleeding, blood transfusion, and sensitization events or debilitating stroke.^3^^,^^6^ The authors suggest that IHM for medical optimization may be particularly beneficial after LVAD placement in patients with pulmonary hypertension, although improvement in outcomes following transplantation have yet to be demonstrated.

Increased adoption of IHM in patients with an LVAD (and advanced heart failure) has the potential to alter the transplant landscape by improving listing candidacy or status justification for patients in outpatient settings. These benefits may potentially lower waitlist time and mortality with earlier transplantation, obviating the need for prolonged hospital admission before cardiac transplantation.

Lambert and Teuteberg^5^ provide a glimpse into the potential future of smart IHM technology linked to automatic changes in LVAD speed to augment cardiac output and optimize central filling pressures. They conclude that evidence to date does not support the routine use of IHM in patients with an LVAD, but as application of remote monitoring increases in heart failure patients, further study is warranted. The value of Big Brother watching our patients with an LVAD remains to be validated.
